# Surgical treatment of rare pediatric cardiac myxomas:12 years clinical experience in a single institution

**DOI:** 10.1186/s12872-023-03255-2

**Published:** 2023-04-28

**Authors:** Shengliang Zhao, Hua Li, Chun Wu, Zhengxia Pan, Gang Wang, Jiangtao Dai

**Affiliations:** 1 Department of Cardio-Thoracic Surgery, Children’s Hospital of Chongqing Medical University, No. 20, Jinyu Avenue, Liangjiang New District, Chongqing, P. R. China; 2 Ministry of Education Key Laboratory of Child Development and Disorders, Chongqing Key laboratory of Pediatrics, National Clinical Research Center for Child Health and Disorders, China International Science and Technology Cooperation base of Child development and Critical Disorders, Chongqing, P. R. China; 3 Department of Thoracic Surgery, Second Affiliated Hospital of Army Medical University, Chongqing, P. R. China

**Keywords:** Cardiac myxomas, Primary cardiac tumor, Cardiac surgery, Pediatric

## Abstract

**Background:**

Primary cardiac tumors are rare, and cardiac myxoma (CM) accounts for the majority of these tumors. Most of the reports in the literature are case reports. This study summarizes our clinical experience in the surgical treatment of CM over the past 12 years.

**Methods:**

We retrospectively analyzed the clinical data of 23 children with CM(8 boys, 15 girls; median age: 8.92 months, range: 2 years 5 months-12 years 9 months; body weight: 11-45 kg, median body weight: 28.21 kg) admitted to our hospital in the previous 12 years, and we statistically analyzed their clinical manifestations and surgical methods.

**Results:**

23 cases underwent myxoma excision under cardiopulmonary bypass(CPB). The follow-up period was 0.2 to 12.6 years (mean:7.2 years). Two patients could not be traced, and the follow-up completion rate was 91.30%. One patient (4.35%) died of myocardial infarction early after surgery with low continuous cardiac output. There were no cerebral embolism, acute heart failure, atrioventricular block and other related complications in 19 cases. A patient with cerebral infarction complicated with right hemiplegia recovered well after rehabilitation treatment. There was no recurrence of CM in 19 cases and all patients recovered after surgery. One patient relapsed 5 years after surgery, and no tumor recurrence was observed after the second surgery. Among the 20 long-term survivors, 13 (65.00%) were NYHA Class I patients and 7(35.00%) were NYHA Class II patients.

**Conclusions:**

Although CM in children is rare, it may cause cerebral infarction and other multi-organ embolism. Once CM is found and removed as soon as possible, it can reduce serious complications. If the complete resection is possible, surgery provides better palliation. Follow-up echocardiographic should be paid attention to after surgery.

## Background

Primary cardiac tumors are rare. It is estimated that the annual incidence rate is 0.5-1.0 cases per 1 million people [1]. About 90% of primary cardiac tumors are benign. The most common benign tumor type of the heart is myxoma, which occurs in the left atrium in 75–85% of cases [2, 3]. CM has the risk of thrombosis caused by tumor thrombus shedding, causing hemodynamic abnormalities and endangering the life safety of patients. Although symptoms of intracardiac obstruction are common in CM patients, this tumor has the risk of systemic embolism, cerebral or peripheral infarction, syncope and sudden death [4]. It should be discovered and operated as early as possible. Surgical resection is an effective treatment [1–4]. Most cases reported in the literature are adults. In the past 12 years, 23 children with rare CM were treated in our hospital. Here, we shared our experience in the diagnosis and treatment of childhood CM.

## Materials and methods

### Patients

We retrospectively analysed the clinical data of 23 children with CM admitted to the Department of Cardiothoracic Surgery, Children’s Hospital of Chongqing Medical University from January 2010 to January 2022. The study protocol is performed in accordance with the relevant guidelines and regulations. The guidelines and regulations followed in this study are the use of standard aortic and separate vena cava catheters. Myocardial protection was achieved by cold crystalloid cardioplegia [4]. 

## Operative techniques

Surgery was performed shortly after the diagnosis of CM to avoid the potential life-threatening complications such as valvular obstruction or embolic phenomena. All patients were approached via median sternotomy with the aid of CPB and mild hypothermia. Standard aortic and separate vena caval cannulation was used. Myocardial protection was achieved by cold crystalloid cardioplegia [4]. Cardiac manipulation was avoided until the aorta was cross-clamped to avoid tumour fragmentation and systemic embolization. Thirteen cases diagnosed as CMs underwent surgical treatment within one week, six cases underwent surgery within one month, and four cases underwent surgery for six months to one year. In myxomas of the right atrium, the main pulmonary artery was cross-clamped as well to prevent possible embolization of tumour fragments into the pulmonary arteries. Intra-cardiac access was achieved through left atrial, right atrial, bi-atrial and right atrial trans-septal approaches according to the tumour location. For patients with left atrial myxoma, the right atrial septum incision was made, the right atrium was diagramed, the atrial septum was cut along the foramen ovale, the tumor was elevated to the right atrium, and the tumor tissue was completely removed. The right atrium, right ventricle, left ventricle, mitral valve and tricuspid valve were investigated during the operation. After the tumor was removed, the heart cavity was repeatedly flushed with normal saline.

## Statistical analyses

All the collected data were statistically analyzed using SPSS 22.0 software. Continuous variables were represented by mean ± standard deviation, and categorical variables were presented as counts and percentages.

## Results

Demographic data and clinical characteristics are detailed in Table 1. There were 8(34.78%)males and 15(65.22%) females, aged 2 years and 5 months-12 years and 9 months, with a median age of 8.92 years and a body weight of 11-45 kg (28.21 ± 13.42 kg). There were 15(65.22%) left atrial myxomas, 7(30.43%) right atrial myxomas and 1 (4.35%) right ventricular myxomas. None of the patient had familial CM. The duration of symptoms ranged from 2 days to 1 year. Twelve patients (52.17%) were in New York Heart Association (NYHA) Class III, 9 (39.13%) in NYHA II and 2 (8.70%) presented in NYHA class IV. The clinical manifestations were cough, shortness of breath, and palpitations in 9 cases, weakness in upper and lower limbs with aphasia in 2 cases, dizziness and fainting in 3 cases, and a significant decrease in exercise tolerance in 6 cases. One patient presented with superior vena cava obstruction syndrome, edema of the head, face, neck and upper limbs, and one patient had cerebral infarction with hemiplegia of the right limb. The duration of clinical symptoms in these patients ranges from one week to over a year. See Table 1 for details. One patient was found without any clinical symptoms by routine physical examination.

**Table 1:** Clinical characteristics of the cardiac myxoma patients (n = 23)

General information	N (%)
Gender (male / female)	8(34.78%)/ 15(65.22%)
Age, years (mean ± SD)	2.42–12.75 (8.92 ± 3.97)
Weight (kg)	11- 45 (28.21 ± 13.42)
Location:	
Left atrium Right atrium Right ventricle	15 (65.22%) 7(30.43%) 1 (4.35%)
NYHA class:	
NYHA II NYHA III NYHA IV	9 (39.13%) 12(52.17%) 2 (8.70%)
Clinical manifestations:	
Asymptomatic	1 (4.35%)
Cough, shortness of breath, and palpitations	9 (39.13%)
Weakness in upper and lower limbs with aphasia	2 (8.70%)
Dizziness and fainting	3 (13.04%)
Decrease in exercise tolerance	6 (26.09%)
Superior vena cava obstruction syndrome	1 (4.35%)
Cerebral infarction with hemiplegia	1 (4.35%)
The duration of symptoms:	
Within 1 week January to March March to June June to 1 year Over 1 year	8(34.78%) 2(8.70%) 6(26.09%) 4(17.39%) 3(13.04%)
Physical examination:	
simple diastolic murmurs	18(78.26%)
simple systolic murmurs	5(21.74%)
Electrocardiogram:	
Sinus tachycardia	7(30.43%)
Atrial fifibrillation	9(39.13%)
Abnormal P wave	2(8.70%)
T wave change	1(4.35%)
Normal	4 (17.39%)
The site of origin of the CM:	
Interatrial septum	10 (43.48%)
Lateral atrial wall	6 (26.09%)
Right superior pulmonary vein	2 (8.70%)
Mitral valve	3 (13.04%)
Tricuspid valve	1(4.35%)
Right ventricle	1(4.35%)
The tumor size (Shortest diameter×longest diameter):	
< 2 × 3 cm	6(26.09%)
2 × 3 cm-3 × 5 cm	8(34.78%)
3 × 5 cm-5 × 7 cm	7(30.43%)
> 7 × 8 cm	2(8.70%)

**Fig. 1:**
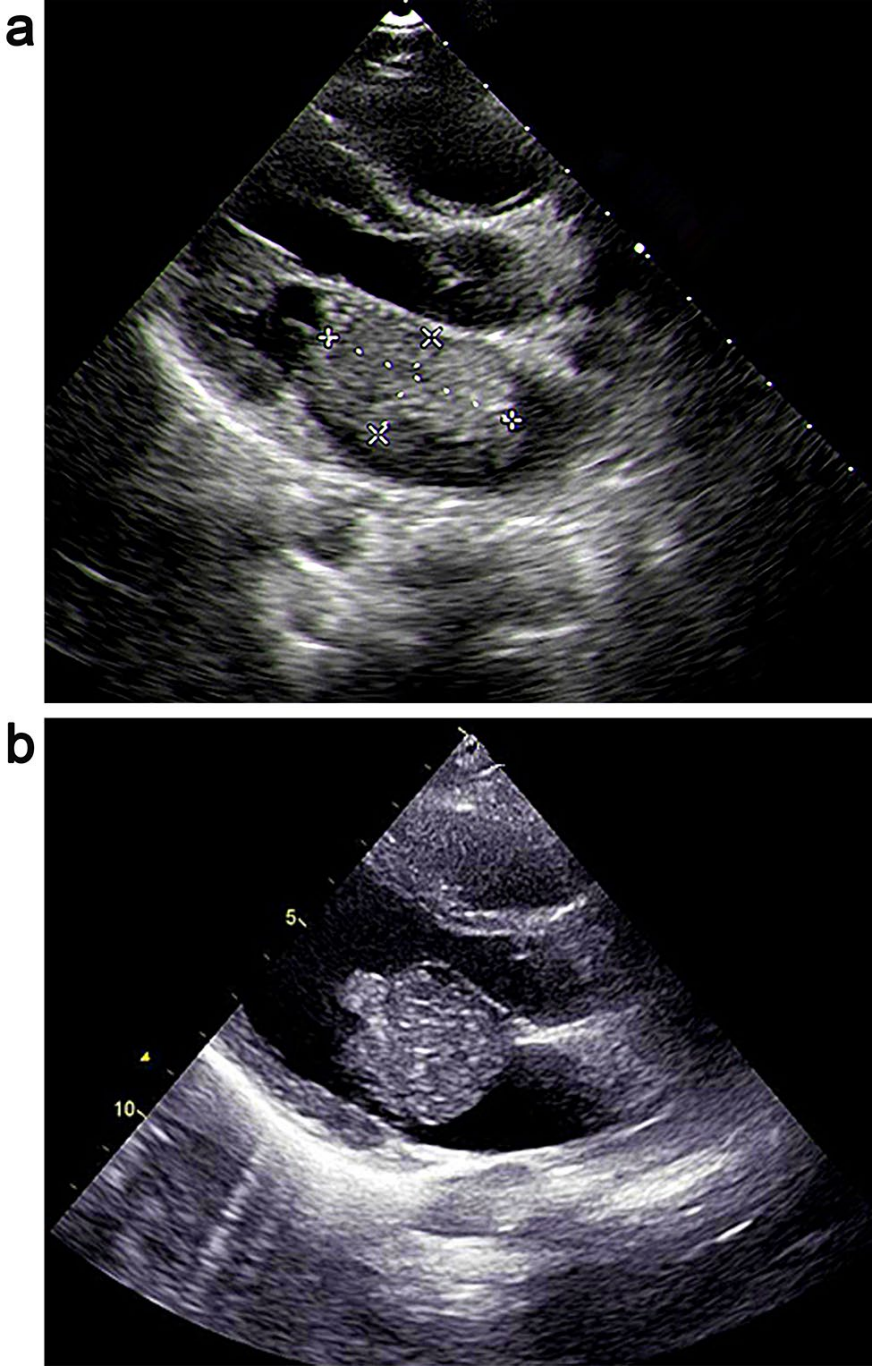
a, b TTE showed a pedunculated mass attached to the atrial septum

**Fig. 2:**
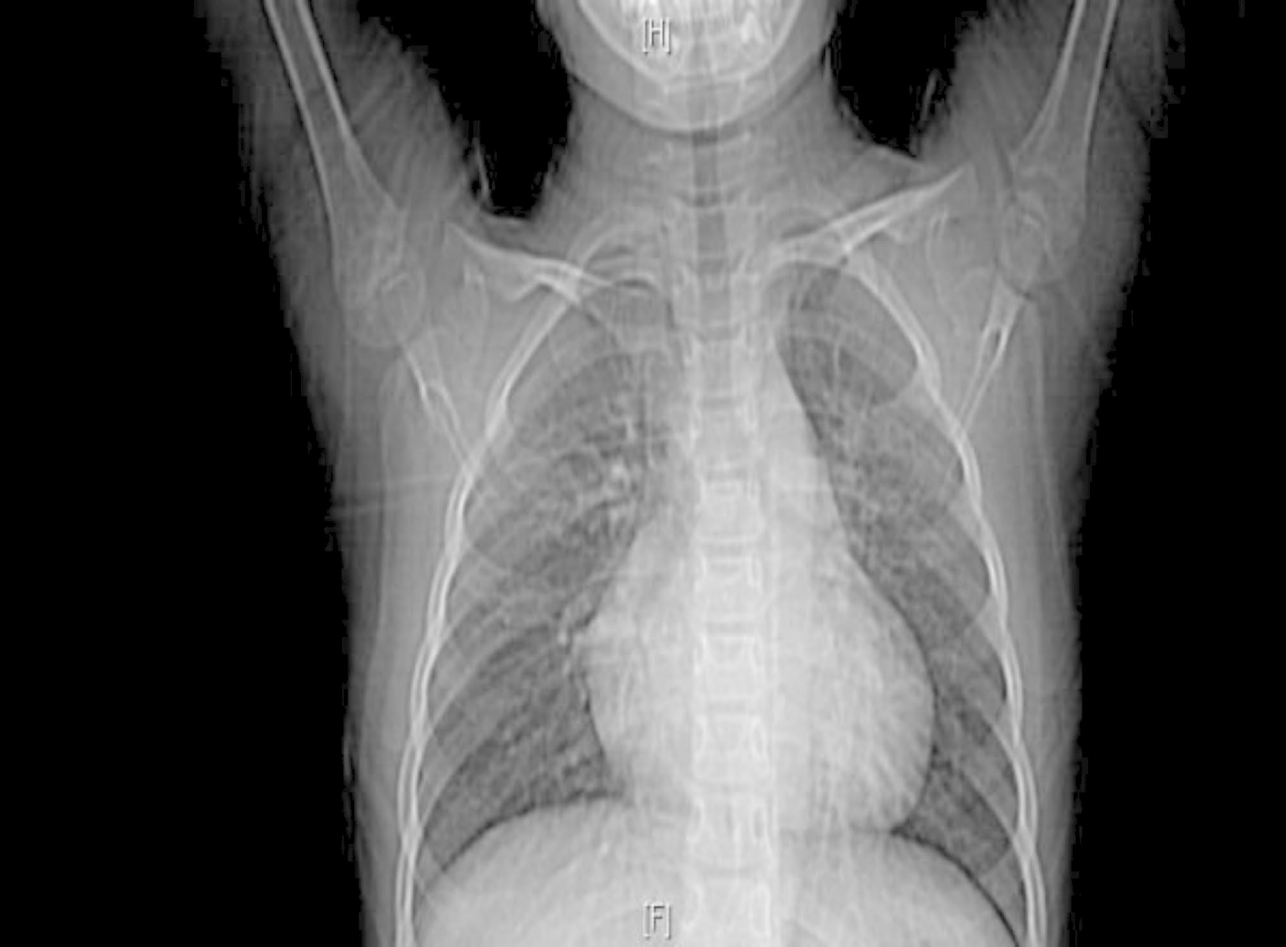
Preoperative chest X-ray revealed a large heart in the patient with cardiac myxoma

There were 19 cases with abnormal electrocardiogram, 7 cases with sinus tachycardia, 9 cases with atrial fifibrillation, 2 cases with abnormal P wave,1 case with T wave change, and 4 cases without obvious abnormality. Physical examination: 23 cases had cardiac murmurs at the apex, including 18 cases of simple diastolic murmurs and 5 cases of simple systolic murmurs. All the patients were diagnosed by echocardiography. Echocardiogram showed that there was abnormal echo light mass in the atrium with unclear boundary, which could move along with the contraction and relaxation of the heart. We can clearly observe a large mass of uniform density attached to the atrial wall. See Fig. 1 for details. Preoperative chest X-ray revealed a large heart in the patient with CM. See Fig. 2 for details.

**Fig. 3:**
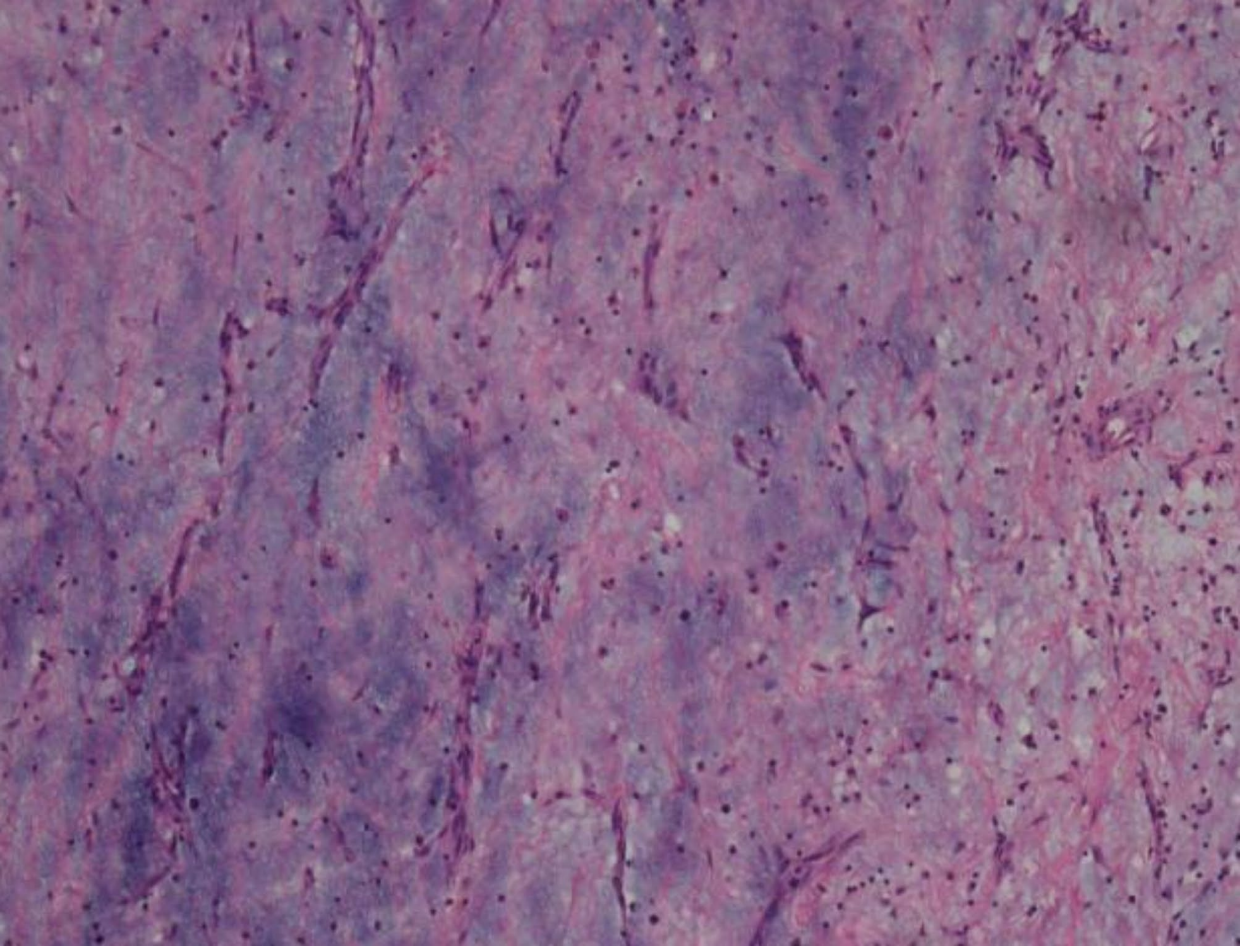
Histology of the excised tumour demonstrating uniform spindled and stellate cells with pale eosinophilic cytoplasm consistent with a diagnosis of cardiac myxoma.(f×200)

Regarding the site of origin of the CM, intraoperative findings revealed that 10 (43.48%) myxomas arose from the interatrial septum, 6 (26.09%) were attached to the lateral atrial wall, 2 (8.70%) were attached to the right superior pulmonary vein, 3 (13.04%) were attached to the mitral valve, 1(4.35%) was attached to the tricuspid valve, and 1(4.35%) was attached to the lateral wall of the right ventricle. The tumor size ranged from 2 × 3 cm to 7 × 8 cm and weighed 5-45 g. The external appearance of the tumors was usually that of a gelatinous, sessile or pedunculated mass. Histology of the excised tumour demonstrating uniform spindled and stellate cells with pale eosinophilic cytoplasm consistent with a diagnosis of CM. See Fig. 3 for details.

**Fig. 4:**
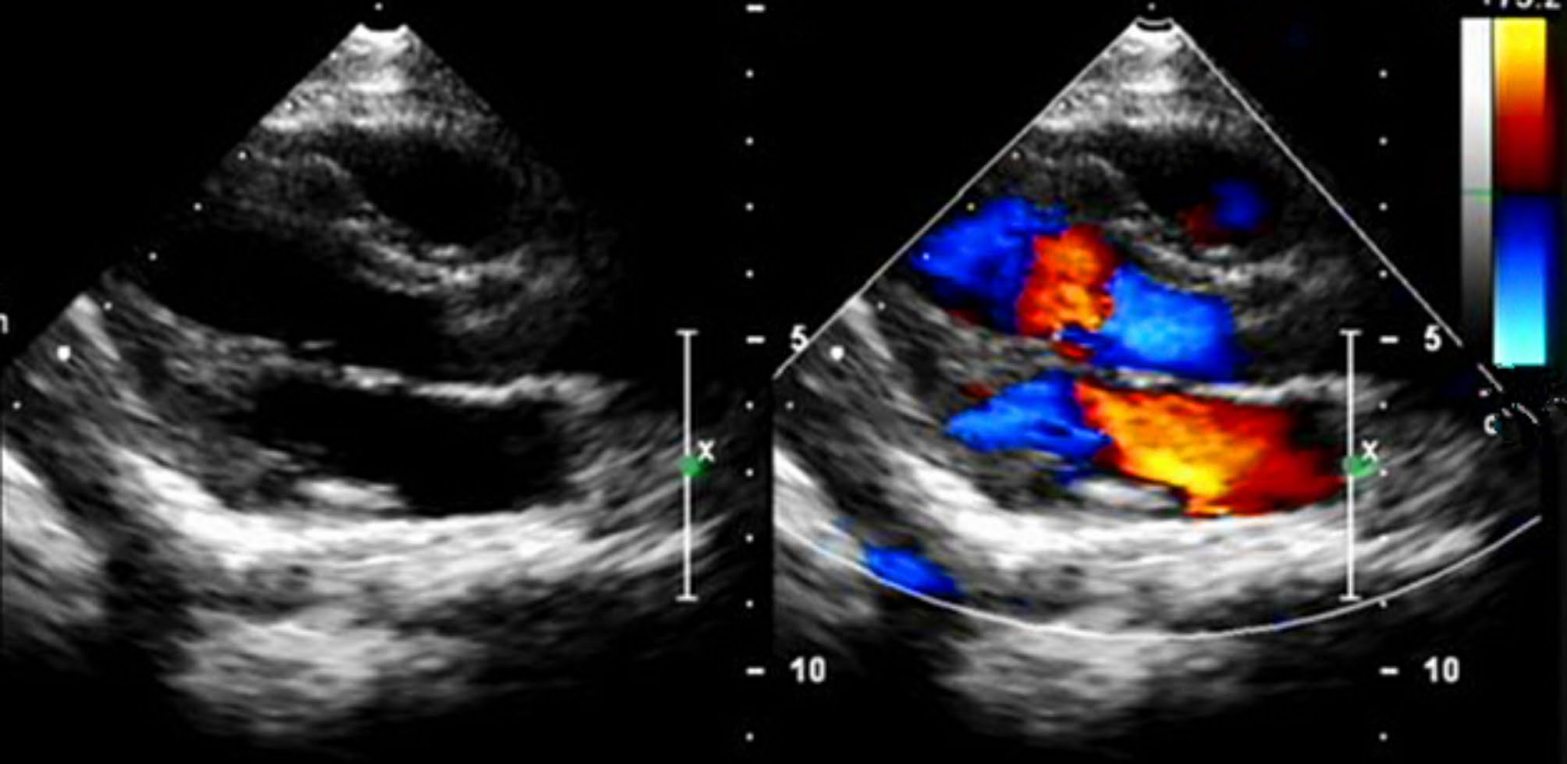
After the operation, TTE showed that the space-occupying mass in the cardiac had disappeared

In our study, the most commonly used method was the right atrial trans-septal approach, followed by the right and left atrium. CM resection under CPB was performed in 23 cases, foramen ovale suture was performed in 16 cases, pericardial repair was performed in 7 cases with large atrial septal defect, mitral valve repair was performed in 3 cases, and tricuspid ring contraction was performed in 2 cases. See Table 2 for details. The mean time of aortic occlusion was 40.12 min (38–43 min), the mean time of CPB was 69.67 min (65–72 min), and the mean time of operation was 205.36 min (155–265 min). Postoperatively, TTE showed the absence of a space-occupying mass in the heart. See Fig. 4 for details. In most patients, the course of postoperative treatment is smooth. A few patients had some complications during hospitalization. There were 8 cases of postoperative arrhythmia and 4 cases of postoperative low cardiac output syndrome.

**Table 2:** The treatment methods of the cardiac myxoma patients (n = 23)

The treatment methods	N (%)
The right atrial trans-septal approach	16(69.57%)
Right and left atrium approach	7(30.43%)
Foramen ovale suture	16(69.57%)
Pericardial repair	7 (30.43%)
Mitral valve repair	3 (13.04%)
Tricuspid ring contraction	2 (8.70%)

Survivors were followed up by questionnaire and telephone contact. The follow-up period was 0.2 to 12.6 years (mean:7.2 years). Two patients could not be traced, and the follow-up completion rate was 91.30%. One patient (4.35%) died of myocardial infarction early after surgery with low continuous cardiac output. There were no cerebral embolism, acute heart failure, atrioventricular block and other related complications in 19 cases. A patient with cerebral infarction complicated with right hemiplegia recovered well after rehabilitation treatment. There was no recurrence of CM in 19 cases and all patients recovered after surgery. One patient relapsed 5 years after surgery, and no tumor recurrence was observed after the second surgery. Among the 20 long-term survivors, 13 (65.00%) were NYHA Class I patients and 7(35.00%) were NYHA Class II patients.

## Discussion

Cardiac myxoma (CM) is a rare primary benign tumor, accounting for 75% of the total number of women [5]. The most common site is the left atrium, and the vertebral pedicle is often located in the foramen ovale of the atrial septum [5]. About 90% of primary cardiac tumors are benign. According to the literature, the average growth rate of CM is 0.49 cm/month, but the actual growth rate(The rate of multiplication of tumor size)is still a controversial issue [6]. A total of 23 cases were included in this study, including 8 males and 15 females, including 7 right CMs and 16 left CMs, consistent with the literature. The cause of CM is unknown. Some studies have shown that CM originates from pluripotent cardiac stem cells [7]. Some studies have shown that CM may be familial and may be an autosomal recessive disease in some families [8]. It is not clear whether the environment or other factors are related to the disease [8]. In our study, they can be found in any chamber of the heart, but the frequency is more common in Left atrium (LA)(65.22%). Most CM originated from the atrial septum (43.48%), but about 39.13% originated from the outer wall of the atrium or other regions such as the mitral valve. In our study, only a few CM (17.40%) originated from the right superior pulmonary vein, tricuspid valve and right ventricle.

The clinical symptoms of CM are not obvious, and they are often found on physical examination or after acute cerebral infarction. Tumor activity can block the atrioventricular valve and cause obstruction of blood flow, causing abnormal hemodynamics, and severely causing sudden death. At the same time, the tumor is fragile and easy to fall off, which can lead to embolism.Therefore, it should be detected early and operated early, and surgery is an effective treatment method [9]. CM may present with various symptoms depending on their location, size, and functional derangements caused by the tumour. The main symptoms of the children in this study included palpitations, cough, syncope and shortness of breath. Neurological symptoms are the second most common finding. These symptoms may be caused by thromboembolic complications of CM. In 30–40% of cases, CM located in left atrium has a risk of systemic embolism [10]. In this group, 2 cases were treated in neurology department due to lower limb muscle strength in the early stage, but did not visit our Department of Cardio-Thoracic Surgery. One patient went to the neurosurgery department for diagnosis of cerebral infarction and cerebral vasodilation, and one patient was transferred to the respiratory department for cough, expectoration, fever and shortness of breath. One patient died of myocardial infarction early after surgery with low continuous cardiac output.Therefore, the early diagnosis of CM is very difficult, and it is easy to be misdiagnosed as other diseases.

Echocardiography is the most important diagnostic method at present; It is non-invasive, and there is no risk of tumor embolism. Echocardiography can easily determine the relationship between the location, size and quality and the intracardiac components [11, 12]. In our series of studies, all CM was initially displayed by transthoracic echocardiography (TTE) and then supplemented by intraoperative transesophageal echocardiography (TEE).

We understand that the tissue of CM in children is fragile, and those with incomplete capsules are more likely to be broken.Therefore, the operation should be gentle during the operation to avoid turning and squeezing the heart. The normal atrial septal tissue where the tumor is attached should be clamped when the tumor is resected. We lift the tumor completely out of the heart cavity to prevent the tumor from breaking. We completely remove the tumor and attached tissues during the operation, and carefully check for any defects after the tumor is removed to prevent tumor fragments from being left. Large resections and patch reconstructions close to the conduction system or mitral valve are technically difficult and dangerous, so resection of tumoural tissues close to these structures may be limited to the subendocardial level. Following the tumor removal from the field, the area should be liberally irrigated, suctioned, and inspected for loose fragments. In this study, 7 cases underwent pericardial repair due to large atrial septal defect, and 3 cases underwent mitral valvuloplasty due to the large base of the tumor, which can easily damage the left atrium and affect the structure of the mitral valve. Two cases with a large tumor expanded the tricuspid annulus, so tricuspid annulus contraction was performed.

The malignant potential of CM was reported in previous studies. Local recurrence and remote metastasis may develop in cases with incomplete resection, intraoperative implantation or embolization, and in cases with malignant transformation potential. Approximately 25% of primary cardiac tumors are malignant, about 75% of which are sarcomas [13]. If the complete resection is possible, surgery provides better palliation and can possibly double survival. In our series, the pathological findings of 23 CM were benign in nature. The possibility of recurrence of CM after surgical resection is extremely low, less than 5%, and only a few cases have been recorded [14]. In this study, there was 1 case of recurrence 5 years after surgery. The reasons for the recurrence were: incomplete resection of the tumor and residual tumor, and possible tumor implantation in the heart.Therefore, we should pay attention to the postoperative follow-up of patients with CM. The hospital mortality after an excision of CM is reportedly about 5% [15], while in this series it was 4.35%. This is consistent with the literature reports.

So far, some studies have found that 34 protein markers are involved in the histogenesis and development of CM [16]. Some potential therapeutic targets such as MUC1, EGFR, FOS and MMP9 have been identified as key targets. This means that drug treatment of CM may become a reality.However, there is no relevant report in clinical practice.Therefore, early diagnosis of CM is particularly important to provide more treatment options. Surgical treatment should be carried out as soon as possible, because as many as 8% of patients may die of obstruction or embolism while waiting for surgery [17]. In the clinical practice center with mature minimally invasive experience, thoracoscopic resection is the first choice for surgical treatment of CM [18]. However, due to the narrow operation space of thoracoscopic surgery and the need for special equipment and peripheral CPB, clinical practice is difficult, and the safety and clinical effect of thoracoscopic CM resection are still in doubt. Due to the differences in surgical field exposure and observation methods, cardiac tumor resection is incomplete, tumor tissue is easy to fall off during surgery, and it is difficult to completely resect during surgery, which may lead to postoperative recurrence or increase the risk of systemic embolism [19]. 

## Conclusions

Although CM in children is rare, it may cause cerebral infarction and other multi-organ embolism. Once CM is found and removed as soon as possible, it can reduce serious complications. If the complete resection is possible, surgery provides better palliation.Follow-up echocardiographic should be paid attention to after surgery.

## Data Availability

The datasets used and analyzed during the current study are available from the corresponding author upon reasonable request.

## List of abbreviations

CM: cardiac myxomas
NYHA: New York Heart Association
TEE: transesophageal echocardiography
TTE: transthoracic echocardiography.
CPB: cardiopulmonary bypass
LA: Left atrium
